# Extending calla lily vase life using silver nitrate, silver nanoparticles, and chlorogenic acid: a novel strategy to sustain xylem conductivity and antioxidant defense

**DOI:** 10.1186/s12870-026-09317-9

**Published:** 2026-07-02

**Authors:** Iman Mohamed El-Sayed, Rasha Ahmed El-Ziat, Eman Zaky Othman

**Affiliations:** 1 Department of Ornamental Plants and Woody Trees, National Research Centre (NRC), Giza, 12622 Egypt; 2 Ornamental Horticulture Department, Faculty of Agriculture, Cairo University, Giza, 12613 Egypt

**Keywords:** Antioxidant enzymes, Eco-friendly postharvest technology, Microbial proliferation, Senescence, Water relations, *Zantedeschia aethiopica*

## Abstract

The postharvest longevity of *Zantedeschia aethiopica* (cut calla lily) flowers is often limited by rapid microbial proliferation and water loss, leading to early wilting and reduced aesthetic quality. This research aimed to evaluate silver nitrate (AgNO₃), silver nanoparticles (AgNPs), and chlorogenic acid (CGA) as preservative agents to maintain physiological and biochemical quality and extend vase life in cut calla lily flowers. Each treatment was applied individually in a completely randomized design (CRD) with three replicates of seven inflorescences each (totaling 21 inflorescences per treatment), using seven vase solutions supplemented with 15 mmol L⁻¹ sucrose: distilled water (control), AgNO₃ at 50 and 100 mg L⁻¹, AgNPs at 15 and 30 mg L⁻¹, and CGA at 15 and 30 mg L⁻¹. Treatments were evaluated at nine predetermined time points, after which their effects were assessed on vase life, water uptake, relative fresh weight (RFW%), relative chlorophyll index (SPAD), total soluble sugars (TSS), protein content, total phenolic content (TPC), hydrogen peroxide (H₂O₂), malondialdehyde (MDA), microbial proliferation at the stem base, and the activities of antioxidant enzymes catalase (CAT) and superoxide dismutase (SOD). Data were analyzed using one-way ANOVA followed by Duncan’s multiple range test (*p ≤* 0.05). These findings indicate that AgNPs and CGA significantly extended the shelf life of cut calla lily flowers, with optimal concentrations of 30 mg L⁻¹ CGA and 30 mg L⁻¹ AgNPs. The highest RFW% (101.7 and 101.02%) was obtained from treatment with 30 mg L⁻¹ CGA on day 5 in both seasons. These treatments improved water uptake, reduced water loss, inhibited microbial activity at the stem base, and decreased microbial blockages in the xylem for up to 11 days. Furthermore, treatment with 30 mg L⁻¹ CGA resulted in the highest values of relative chlorophyll index (66.05 and 65.08 SPAD in the first and second seasons, respectively), total protein content (26.09 and 23.08 mg g⁻¹ FW, respectively), and soluble carbohydrates (2.47% and 2.40%, respectively). In addition, this treatment reduced H₂O₂ and MDA accumulation and enhanced antioxidant enzyme activities. Briefly, our results highlight the use of CGA and AgNPs, as well as the potential of incorporating natural antioxidants as a sustainable alternative to conventional preservatives in postharvest floral management.

## Introduction

*Zantedeschia aethiopica* L. Spreng, commonly known as calla lily or arum lily, is a perennial rhizomatous flowering plant in the family Araceae native to southern Africa [1]. Owing to its distinctive floral architecture, abundant flower production, and extended blooming period, this species has gained widespread popularity as a high-value ornamental plant in the global floriculture market [2, 3]. The inflorescence is characterized by a conspicuous tubular white spathe, which partially encloses a central yellow spadix bearing unisexual flowers [4]. Beyond its unique developmental features, the calla lily’s elegant floral structure has made it a popular choice for wedding, funeral, and other formal event floral arrangements [5]. Since the 1990s, global demand for calla lilies has increased significantly, driven by advancements in postharvest handling, cooling, and transportation technologies, which have enabled year-round availability [5]. The species is currently cultivated commercially in over twenty-five countries, supplying major markets in Europe, Asia, and North America [6]. Despite its high commercial value, the yield of premium-quality flowers per plant remains relatively low, posing a significant constraint on production efficiency and overall profitability [7]. Moreover, cut calla lily flowers exhibit inherently short postharvest longevity and are difficult to maintain in optimal visual quality when harvested at full spathe opening. Under such conditions, their vase life is typically limited to 6–7 days, after which rapid deterioration occurs. This highlights the need for effective postharvest strategies to enhance longevity and preserve ornamental quality. In addition, cut calla lily flowers face considerable postharvest challenges, particularly under the constraints of long-distance transportation and extended display periods [8]. One of the major causes of early wilting is impaired water uptake due to xylem blockage, which may result from bacterial proliferation, air embolism, or the accumulation of inhibitory substances such as suberin and lignin in the vascular tissue [9].

Earlier research has demonstrated that the use of chemical preservatives, such as antimicrobial agents, ethylene inhibitors, and carbohydrate supplements, can significantly enhance water uptake, delay senescence, and preserve flower quality [9]. Among these, silver nitrate (AgNO₃) is highly effective due to its potent antimicrobial and anti-ethylene properties, resulting in improved fresh weight, flower diameter, and vase life [10]. However, environmental and health concerns, along with high costs, have limited their practical application [11], prompting the investigation of silver nanoparticles (AgNPs) as a safer and potentially more efficient alternative [12]. AgNPs possess unique physicochemical properties and exhibit strong antimicrobial activity. They can also move through xylem vessels, minimizing vascular blockage and enhancing water uptake. Additionally, they reduce transpiration by regulating aquaporins and stomatal behavior [13]. Despite these advantages, the potential long-term environmental impacts and phytotoxic effects of AgNPs require further investigation before widespread application [14, 15].

In this context, natural compounds such as chlorogenic acid (CGA) have emerged as promising eco-friendly alternatives. CGA is a plant-derived phenolic compound with well-documented antioxidant, antimicrobial, and stress-mitigating properties [16]. It plays a key role in the regulation of plant metabolism, enhances tolerance to abiotic stress, delays senescence, and improves membrane integrity by reducing electrolyte leakage and the accumulation of reactive oxygen species (ROS) [17]. Furthermore, CGA stimulates antioxidant enzyme activity and modulates energy and phenylpropanoid metabolism, highlighting its potential for postharvest applications [18, 19]. However, its direct application in cut flower preservation remains underexplored. The global demand for ornamental flowers has increased steadily, with the flower trade expanding from approximately EUR 4.8 billion in 2019 to EUR 5.6 billion in 2021 [20]. This growth underscores the importance of developing effective and sustainable postharvest strategies to reduce losses, maintain quality, and extend flower longevity during distribution and display.

Although CGA has been investigated in postharvest studies of horticultural crops, its application to cut flowers, particularly *Z. aethiopica*, remains limited.

In previous studies, different concentrations of silver nitrate (AgNO₃), silver nanoparticles (AgNPs), and chlorogenic acid (CGA) have been applied to improve flower quality and extend storage and vase life in various horticultural crops. For instance, the application of AgNO₃ has been reported to improve flower quality and prolong vase life in cut roses [21]. In addition, Adam and Eldeeb [22] found that AgNO₃ treatment in carnations reduced bacterial proliferation and extended vase life compared with the control. Furthermore, Juthee et al. [23] showed that AgNPs increased gerbera vase life by > 62% due to their antimicrobial effects. Zhang et al. [24] reported that applying 2.5 µg/mL AgNPs to roses reduced bacterial levels, improved antioxidant systems, and suppressed ethylene production. Maity et al. [25] observed that AgNPs improved vase life and postharvest quality in gladiolus spikes. Moreover, Ilea et al. [26] reported that treating strawberry plants with chlorogenic acid increased the storage period. The effectiveness of AgNO₃ and AgNPs is attributed to their antimicrobial properties, which help maintain the stem conductivity, enhance water balance, and delay postharvest senescence. The stems of cut flowers are particularly prone to microbial growth, leading to xylem blockage, reduced water uptake, and accelerated wilting. In contrast, the application of CGA for extending vase life in cut flowers represents a relatively novel approach. While CGA has been successfully applied in other horticultural crops, few studies have explored its potential in cutting flowers. CGA can alleviate oxidative stress by modulating reactive oxygen species (ROS), providing a promising strategy to enhance postharvest flower quality. AgNPs has been used in only a few studies on calla lilies, or perhaps not at all. Similarly, chlorogenic acid has been used as a preservative on some fruit crops, but there are no studies on its use on cut flowers. Therefore, this study is the first of its kind to explore the use of chlorogenic acid as a natural preservative for cut flowers in general, specifically for calla lilies.

Therefore, this study aimed to evaluate the postharvest effects of silver nitrate (AgNO₃), silver nanoparticles (AgNPs), and chlorogenic acid (CGA) on vase life, physiological responses, and biochemical attributes of cut calla lily flowers. The key parameters assessed included water uptake, relative fresh weight, antioxidant defense activity system, and microbial growth at the stem base. By integrating antimicrobial activity with key physiological and biochemical processes, the present findings provide evidence for the potential of chlorogenic acid as a natural preservative to extend the vase life of cut flowers. This relatively unexplored approach in *Zantedeschia aethiopica* may improve postharvest performance while advancing economically viable, environmentally sustainable practices in floriculture.

## Materials and methods

### Plant material

Calla lily (*Zantedeschia aethiopica* (L.( Spreng.) cut flowers used in this study were obtained from the commercial production farm Floramix Trading & Agriculture Projects, Kafr Hakim, Imbaba, Giza, Egypt (30°04′59″ N, 31°06′54″ E, 19 m above sea level). Flowers were harvested during the first and second experimental seasons (2023 and 2024), in the early morning at the commercial maturity stage, when spathes were fully or sufficiently opened, following the recommendations of [5]. The source plants were uniform in vigor, age, and morphological characteristics, and they were cultivated under standard horticultural practices in a shade-net greenhouse (saran house), with controlled irrigation and fertilization schedules. All selected plants were healthy and free from visible diseases, pests, and mechanical damage to ensure experimental uniformity.

#### Final manuscript.docx

**Fig. 1:**
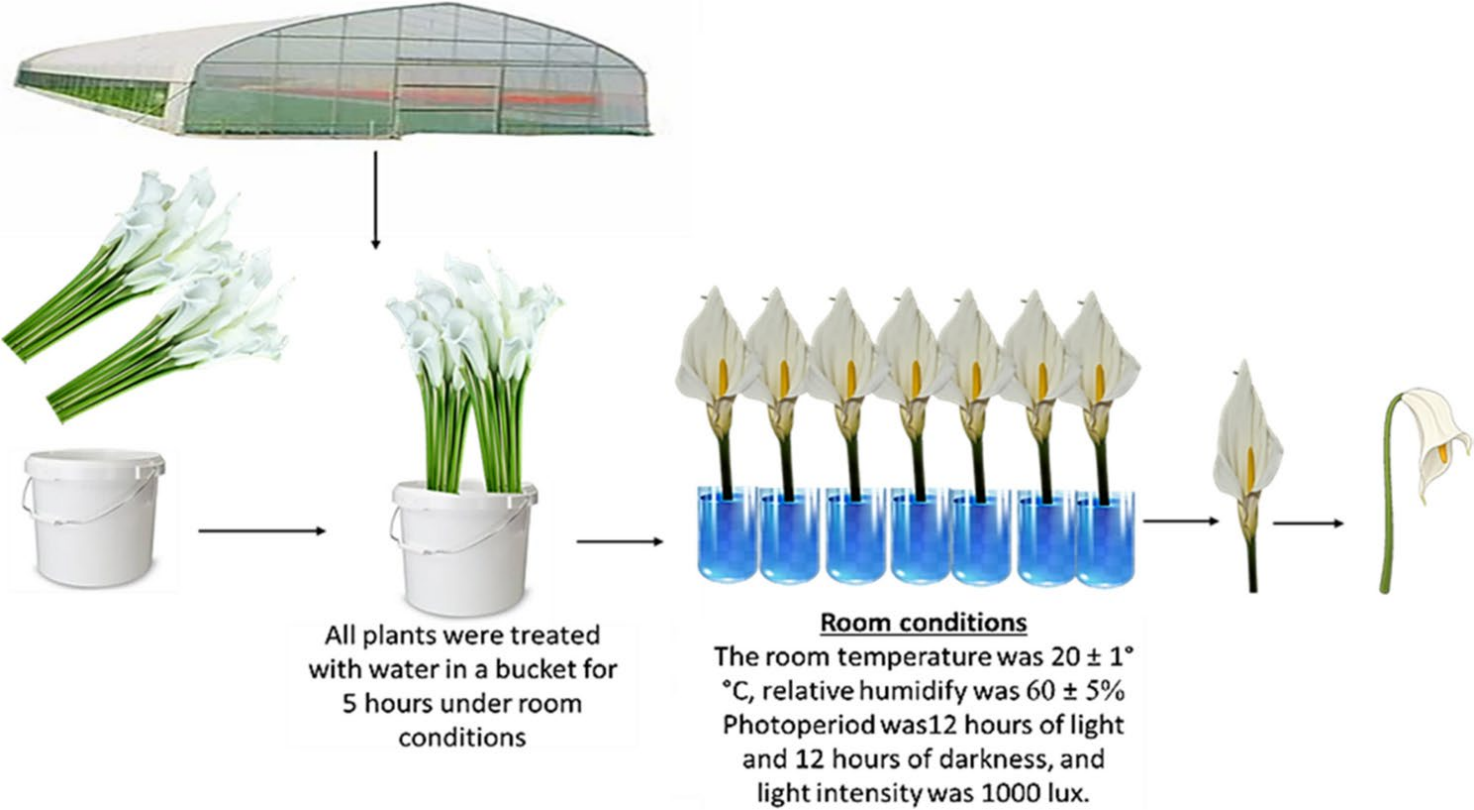
Schematic overview of the experimental design showing flower conditioning and vase treatments. Seven treatments were applied, each consisting of 21 inflorescences arranged in three replicates (n = 3; seven inflorescences per replicate), totaling 147 experimental units

Immediately after harvest, cut flowers were placed in clean containers filled with distilled water and transported to the laboratory of the Ornamental Horticulture Department, Faculty of Agriculture, Cairo University, within 1 h postharvest to minimize postharvest deterioration. Upon arrival, inflorescences were recut under distilled water at the basal end of the stem (2–3 cm above the original cut surface) using a sharp sterile blade at a 45° angle to prevent xylem occlusion and minimize air embolism, thereby ensuring uniform water uptake and hydraulic conductivity. Stems were then standardized to 40 cm in length. Before vase treatments, all flowers were conditioned in distilled water for 5 h at room temperature (22 ± 1 °C and 60 ± 5% relative humidity [RH]) to restore water balance, stabilize metabolism, and facilitate recovery from harvest-induced stress, as illustrated in Fig. 1.

### Experimental details

The experiment was conducted under controlled environmental conditions, with the temperature maintained at 20 ± 1 °C, a relative humidity of 60–65% RH, and a 12-h light/12-h dark photoperiod.

Flowers were placed individually in 600-mL glass vases containing 500 mL of the designated vase solutions. Seven vase treatments were evaluated, each supplemented with 15 mmol L⁻¹ sucrose as a carbon source: distilled water as the control (T1), silver nitrate (AgNO₃) at 50 and 100 mg L⁻¹ (T2 and T3), silver nanoparticles (AgNPs) at 15 and 30 mg L⁻¹ (T4 and T5), and chlorogenic acid (CGA) at 15 and 30 mg L⁻¹ (T6 and T7), as detailed in Table 1. All vase solutions were freshly prepared on the first day of the experiment and were not replaced throughout the evaluation period. Flower quality, water relations, and vase life were monitored at nine predetermined time points: 0, 3, 5, 7, 9, 11, 13, 15, and 17 days. The experiment was conducted using a completely randomized design (CRD), with 21 inflorescences per treatment, divided into three replicates of seven inflorescences each, totaling 147 experimental units.

**Table 1:** Vase solution treatments and concentrations

Vase solution treatment	Concentration
T1: Distilled water (Control)	0 mg L⁻¹
T2: Silver Nitrate (AgNO₃)	50 mg L⁻¹
T3: Silver Nitrate (AgNO₃)	100 mg L⁻¹
T4: Silver nanoparticles (AgNPs)	15 mg L⁻¹
T5: Silver nanoparticles (AgNPs)	30 mg L⁻¹
T6: Chlorogenic acid (CGA)	15 mg L⁻¹
T7: Chlorogenic acid (CGA)	30 mg L⁻¹

### Preparation and use of experimental solutions

Silver nitrate (AgNO₃) and chlorogenic acid (CGA) solutions were obtained from Sigma-Aldrich.

**Fig. 2:**
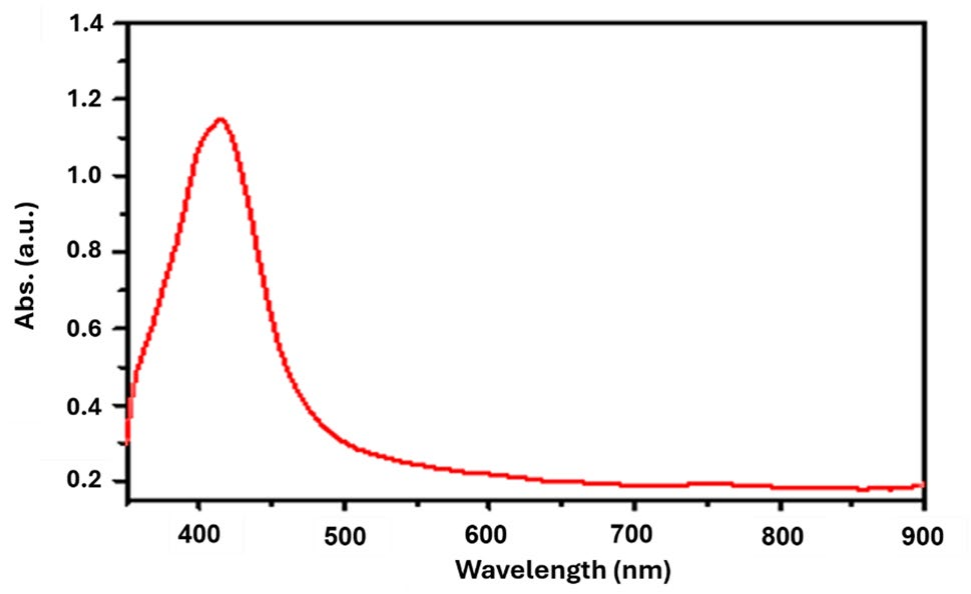
UV–Vis absorption spectrum of the synthesized silver nanoparticles (AgNPs)

**Fig. 3:**
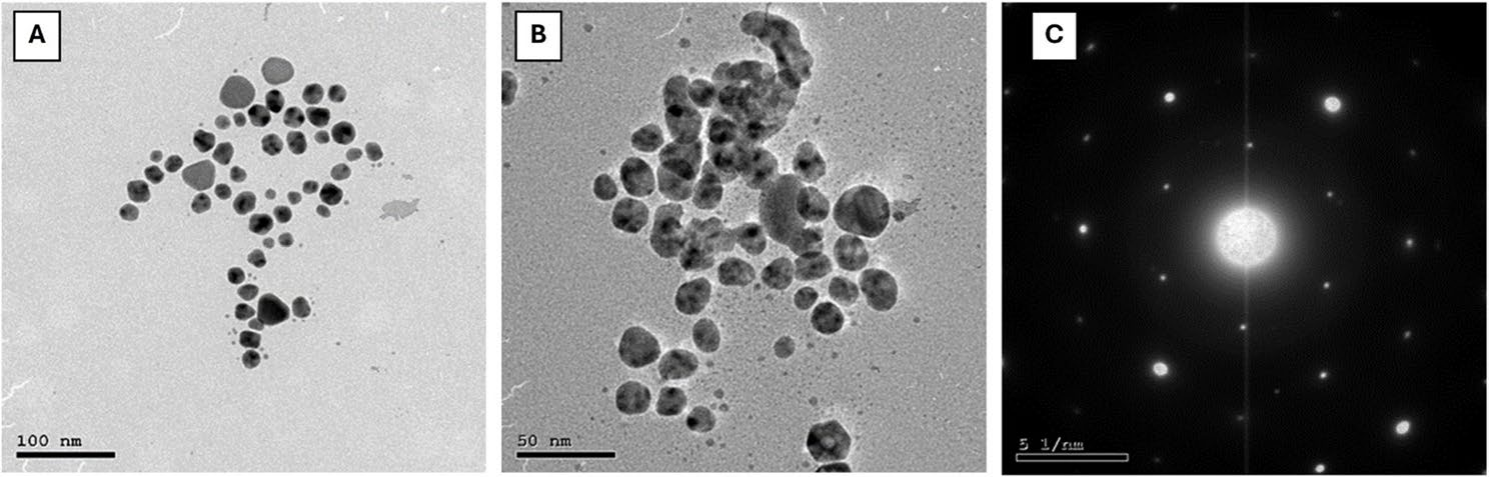
Transmission electron microscopy (TEM) images of silver nanoparticles (AgNPs), including the selected area electron diffraction (SAED) pattern. **A** TEM image, magnification ×100,000, scale bar = 100 nm; (**B**) TEM image, magnification ×200,000, scale bar = 50 nm; (**C**) SAED pattern

Silver nanoparticles (AgNPs) were synthesized via the chemical reduction method described by Lee and Meisel [27]. AgNO₃ served as the Ag⁺ ion precursor, polyvinyl pyrrolidone (PVP) as a stabilizing agent, and sodium borohydride (NaBH₄) acted as a mild reducing agent. The reaction mixture gradually turned grayish-yellow, indicating the formation of AgNPs. The optical properties of the synthesized AgNPs were assessed using UV–Vis absorption spectroscopy (Ocean Optics USB2000 + VIS-NIR spectrophotometer; Fig. 2), while their size and morphology were examined using high-resolution transmission electron microscopy (TEM; JEOL JEM-2100, 200 kV; Fig. 3; Table 2). Fresh AgNPs working solutions were prepared by dispersing the nanoparticles in dimethyl sulfoxide (DMSO) to ensure uniform suspension and particle stability. The dispersions were then thoroughly diluted with distilled water prior to application to minimize any potential effect of DMSO. To minimize microbial contamination and evaporation, all experimental solutions, including AgNPs, AgNO₃, and CGA, were placed in flasks sealed with cotton plugs.

**Table 2:** Physicochemical properties of the synthesized silver nanoparticles (AgNPs)

Property	Description
Appearance (Color)	Deep brown
Appearance (Form)	Powder
Optical Property (UV–Vis λmax)	~ 405 nm
Average Size (TEM)	35 ± 5 nm
Shape (TEM)	Spherical
Stabilizer / Composition	99.5% PVP, 0.5% Ag
Expiration Date	6 months

### Data recorded

#### Vase Life

The vase life of each flower was determined following the method described by [28], in which vase life was defined as the number of days from the initial placement in the vase solution (first day) until the flowers exhibited severe wilting and lost their ornamental value.

#### Water relationship

##### Relative fresh weight (RFW%)

Relative fresh weight (RFW) was determined to assess changes in flower hydration during the vase life period. The initial fresh weight of each scape was recorded at the beginning of the experiment, and subsequent measurements were taken at nine predetermined time points: 0, 3, 5, 7, 9, 11, 13, 15, and 17 days, following the procedure described by Joyce and Jones [29]. The RFW of each scape was calculated using the formula:

$$\mathrm{RFW}\%\;=\;(\mathrm{fresh}\;\mathrm{weight}\;\mathrm{at}\;\mathrm{each}\;\mathrm{measurement}\;/\;\mathrm{initial}\;\mathrm{fresh}\;\mathrm{weight})\;\times\;100.$$

##### Floral water uptake (g flower⁻¹ day⁻¹)

Floral water uptake was measured by recording the weight of the vases containing the preservative solutions (without flowers) at the same nine predetermined time points: 0, 3, 5, 7, 9, 11, 13, 15, and 17 days, following the procedure of Elhindi [30]. Water uptake at each interval was calculated using the following equation:

$$\mathrm{Water}\;\mathrm{uptake}\;(\mathrm g\;\mathrm{flower}^{-1}\;\mathrm{day}^{-1})\;=\;{\mathrm S}_{\mathrm t-1}\;-\;{\mathrm S}_{\mathrm t}$$

where S t is the weight of the vase solution (g) at the current measurement, and St-1 is the weight of the vase solution (g) at the preceding measurement. This measurement reflects the daily consumption of solutions per flower.

##### Water loss (g flower⁻¹ day⁻¹)

Water loss was determined by weighing the vases containing the cut flowers at predetermined time points throughout the vase life period. On each sampling day, the combined weight of the vase and its corresponding flower was recorded, and water loss was calculated according to the method of Lü et al. [31]. The rate of water loss for each interval was then determined using the following equation:

$$\mathrm{Water}\;\mathrm{loss}\;(\mathrm g\;\mathrm{flower}^{-1}\;\mathrm{day}^{-1})\;=\;{\mathrm C}_{\mathrm t-1}\;-\;{\mathrm C}_{\mathrm t}$$

Where *C*_*t*_ represents the combined weight of the cut flower and vase solution (g) at 3, 5, 7, 9, 11, 13, 15, and 17 days, and *C*_*t−1*_ represents the combined weight at the immediately preceding measurements (0, 3, 5, 7, 9, 11, 13, and 15 days, respectively). This parameter quantifies the net loss of solution due to transpiration and evaporation during the evaluation period. 

#### Phytochemical composition of *Z. aethiopica*

All chemicals and reagents used in the following assays were of analytical grade and purchased from Sigma-Aldrich (USA), unless otherwise stated.

##### SPAD value (Relativne chlorophyll idex)

The SPAD value in the spathes of *Z. aethiopica* was measured non-destructively on day 7 of vase life using an SPAD-502 Plus chlorophyll meter (Konica Minolta, Japan). Day 7 was selected because all flowers were still viable at this stage, ensuring consistent and representative measurements. For each flower, three readings were taken along the spathe and averaged to obtain a mean SPAD value. These measurements provide a rapid and reliable estimate of the relative chlorophyll level, reflecting the photosynthetic status and progression of senescence in the cut flowers.

##### Total Soluble Sugars (TSS, %)

The total soluble sugar (TSS) content of *Z. aethiopica* spathes was determined following the method of Irigoyen et al. [32]. Fresh spathe tissue was sampled on day 7 of vase life, and TSS was quantified using the phenol–sulfuric acid method, with absorbance measured at 490 nm using a UV–Vis spectrophotometer (Ocean Optics USB2000 + VIS-NIR, USA). A glucose standard curve was used for calibration, and results were expressed as a percentage of fresh weight (FW %). This measurement reflects the soluble sugar reserves available in the cut flowers at this stage of vase life. 

##### Total soluble protein content (mg g⁻¹ FW)

The total soluble protein content in the spathes of *Z. aethiopica* was determined on day 7 of vase life using the Bradford method [33]. Fresh tissue (0.5 g) was homogenized in 5 mL of 50 mM phosphate buffer (pH 7.0) and centrifuged at 10,000 × g for 15 min (Hettich Universal 320, Hettich, Germany). The protein content in the supernatant was quantified using Bradford reagent, with absorbance measured at 595 nm using a UV–Vis spectrophotometer (Ocean Optics USB2000 + VIS-NIR, USA). A bovine serum albumin (BSA) standard was used for calibration, and results were expressed as mg g⁻¹ fresh weight (FW). This measurement reflects the soluble protein reserves available in the cut flowers at this stage of vase life. 

##### Total phenol content (mg GAE g⁻¹ FW)

The total phenolic content (TPC) in the spathes of *Z. aethiopica* was determined on day 7 of vase life using the Folin–Ciocalteu method [34]. Fresh tissue (0.5 g) was homogenized in 5 mL of 80% methanol and centrifuged at 10,000 × g for 10 min (Hettich Universal 320, Hettich, Germany). An aliquot of the supernatant (0.5 mL) was mixed with 2.5 mL of 10% Folin–Ciocalteu reagent and 2 mL of 7.5% sodium carbonate solution, incubated in the dark at room temperature for 30 min, and absorbance was measured at 765 nm using a UV–Vis spectrophotometer (Ocean Optics USB2000 + VIS-NIR, USA). TPC was calculated from a gallic acid standard curve and expressed as mg GAE (gallic acid equivalent) per g fresh weight (FW). This measurement reflects the antioxidant capacity and phenolic reserves in the cut flowers at this stage of vase life, which are critical for membrane stability, delaying senescence, and enhancing stress tolerance. 

#### Antioxidant enzyme assays

The activities of superoxide dismutase (SOD) and catalase (CAT) were determined in the spathes of *Z. aethiopica* on day 7 of vase life. Fresh spathe tissue (100 mg) was finely ground in a chilled mortar and homogenized in 1 mL of extraction buffer containing 50 mM phosphate-buffered saline (PBS, pH 7.0), 2% polyvinylpyrrolidone (PVP), 1 mM EDTA, and 0.05% Triton X-100. The homogenate was centrifuged at 13,000 rpm for 20 min at 4 °C (Hettich Universal 320, Hettich, Germany), and the resulting supernatant was collected for enzyme assays. Soluble protein content in the enzyme extract was determined using the Lowry method [35] with bovine serum albumin (BSA) as a standard, and absorbance was measured at 640 nm using a UV–Vis spectrophotometer (Ocean Optics USB2000 + VIS-NIR, USA). Enzyme activities were expressed as U mg⁻¹ protein. SOD activity was assayed based on the inhibition of Nitro Blue Tetrazolium (NBT) reduction [36], while CAT activity was determined by monitoring the decomposition of hydrogen peroxide (H₂O₂) [37]. All measurements were performed in triplicate to ensure reproducibility.

#### Assessment of oxidative stress markers

Dry spathe tissue (0.5 g DW) of *Z. aethiopica* was homogenized in ice-cold 0.1% (w/v) trichloroacetic acid (TCA) and centrifuged at 12,000 × g for 15 min at 4 °C (Hettich Universal 320, Hettich, Germany). The resulting supernatant was used to determine oxidative stress markers.

Malondialdehyde (MDA), an indicator of lipid peroxidation, was measured following Heath and Packer [38], with absorbance recorded at 532 and 600 nm using a UV–Vis spectrophotometer (Unico UV-2100, USA). MDA concentration was calculated using an extinction coefficient of 155 mM⁻¹ cm⁻¹ and expressed as nmol g⁻¹ DW. Hydrogen peroxide (H₂O₂), a marker of reactive oxygen species, was quantified according to Alexieva et al. [39] by measuring absorbance at 390 nm using the same spectrophotometer, and results were expressed as µmol g⁻¹ DW. All analyses were performed in triplicate to ensure reliable estimation of lipid peroxidation and oxidative stress during vase life.

#### Averages of bacteria counts (CFU mL^− 1^)

Bacterial growth in the preservative solutions of *Z. aethiopica* was evaluated on day 7 of vase life. One milliliter of each solution was serially diluted with sterile distilled water, and aliquots were plated onto Petri dishes containing 10 mL of sterilized nutrient agar supplemented with peptone. The plates were gently swirled for 5–10 s to ensure uniform distribution and incubated at 30 °C for 36 h. After incubation, bacterial colonies were counted, and results were expressed as CFU mL⁻¹ [40].

This method provides a reliable quantitative assessment of microbial contamination in vase solutions, which can affect the quality and longevity of cut flowers.

#### Scanning Electron Microscopy (SEM)

At the end of the vase life experiment, the xylem vessels at the base of *Z. aethiopica* inflorescence stems were examined to assess bacterial-induced blockages that could impair water transport. Basal stem segments (≈ 2 mm) were excised using sterilized stainless-steel blades, fixed in 2% paraformaldehyde and 2.5% glutaraldehyde for 1 h, and incubated in 0.05 M cacodylate buffer containing 0.001 M CaCl₂ (pH 7.2) at ten times the sample volume. Samples were then washed, stored at 4 °C until further processing, and dehydrated through a graded acetone series (30%, 50%, 70%, and 90% for 10 min each, followed by 100% acetone three times for 10 min). The dehydrated samples were dried, mounted on aluminum stubs, and sputter-coated with a thin layer of gold to enhance conductivity. Prepared samples were examined using a scanning electron microscope (SEM, JEOL JSM-6510LV, JEOL Ltd., Japan) as described by Bozzola and Russell [41]. This analysis provided a detailed visualization of xylem structure and bacterial colonization, offering insights into the role of microbial blockage in reducing hydraulic conductivity and overall cut flower quality during vase life.

### Statistical analysis

The experiment was conducted using a completely randomized design (CRD) with seven treatments, including the control. Each treatment consisted of 21 cut *Z. aethiopica* inflorescences, arranged in three replicates of seven inflorescences each, with one inflorescence per glass vial. Data were analyzed using one-way analysis of variance (ANOVA) at a 5% significance level using SPSS software (version 26, IBM, Armonk, NY, USA). In contrast, correlation analysis, principal component analysis (PCA), and path analysis were conducted using R software (R Software Team, Vienna, Austria) [42].

Because the main objective of this study was to evaluate the effects of postharvest treatments on flower quality and vase life, ANOVA was performed separately for each season to account for seasonal variability. In addition, treatment effects were analyzed independently at each sampling date to ensure comparisons were made at comparable physiological stages, without including time or season as factors in the statistical model. When significant differences were detected, treatment means were separated using Duncan’s multiple range test at *p* ≤ 0.05 [43]. All results are presented as means ± SE of three replicates (*n* = 3). Furthermore, correlation analysis, principal component analysis (PCA), and path analysis were conducted using treatment mean values averaged across the two experimental seasons to explore relationships among measured traits and to determine their relative contributions to vase life.

## Results

### Vase life

**Fig. 4:**
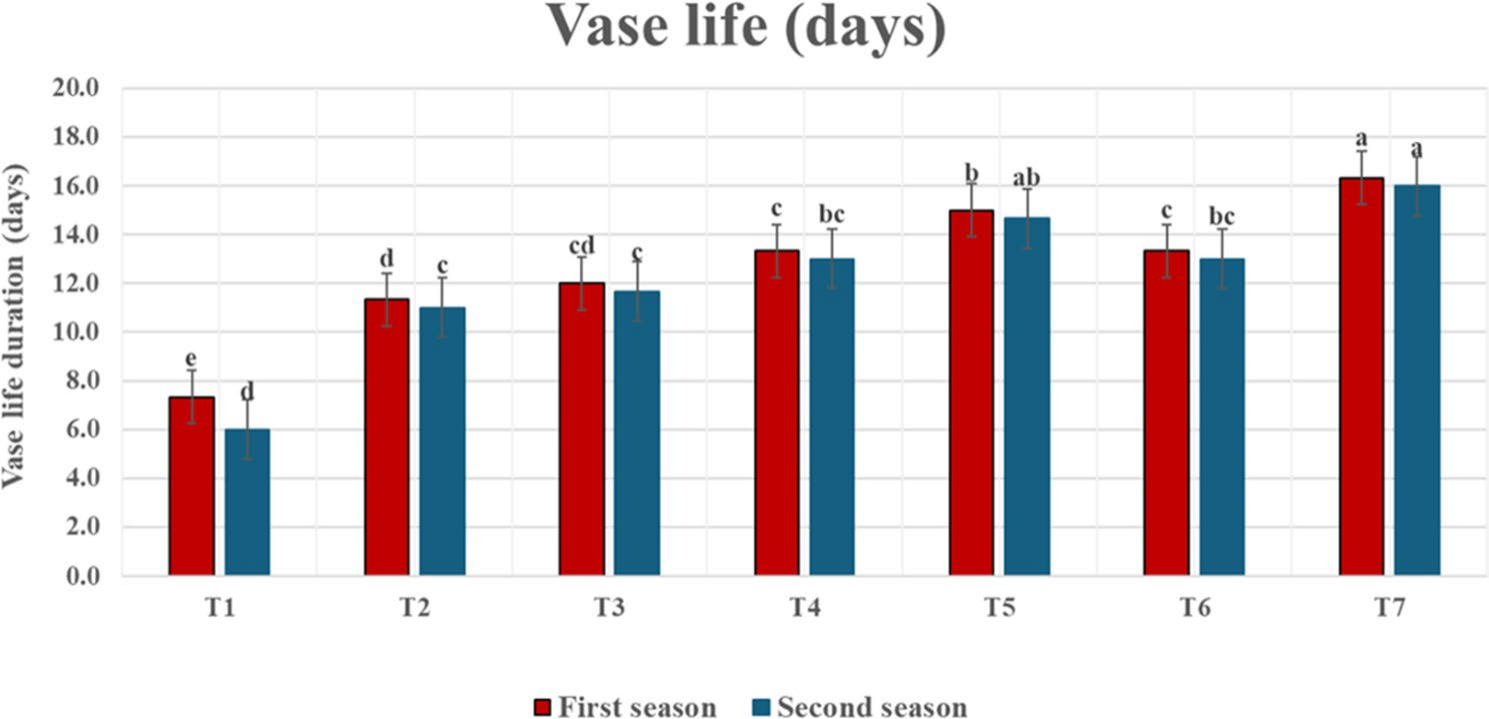
Vase life of *Z. aethiopica* cut flowers treated with AgNO₃, AgNPs, and chlorogenic acid during two experimental seasons. Bars represent means ± SE (*n* = 3) for each season, analyzed separately. Different letters indicate significant differences among treatments within each season according to Duncan’s multiple range test at *p ≤* 0.05. Treatments: T1 = Control; T2 = AgNO₃ 50 mg L⁻¹; T3 = AgNO₃ 100 mg L⁻¹; T4 = AgNPs 15 mg L⁻¹; T5 = AgNPs 30 mg L⁻¹; T6 = CGA 15 mg L⁻¹; T7 = CGA 30 mg L⁻¹

**Fig. 5:**
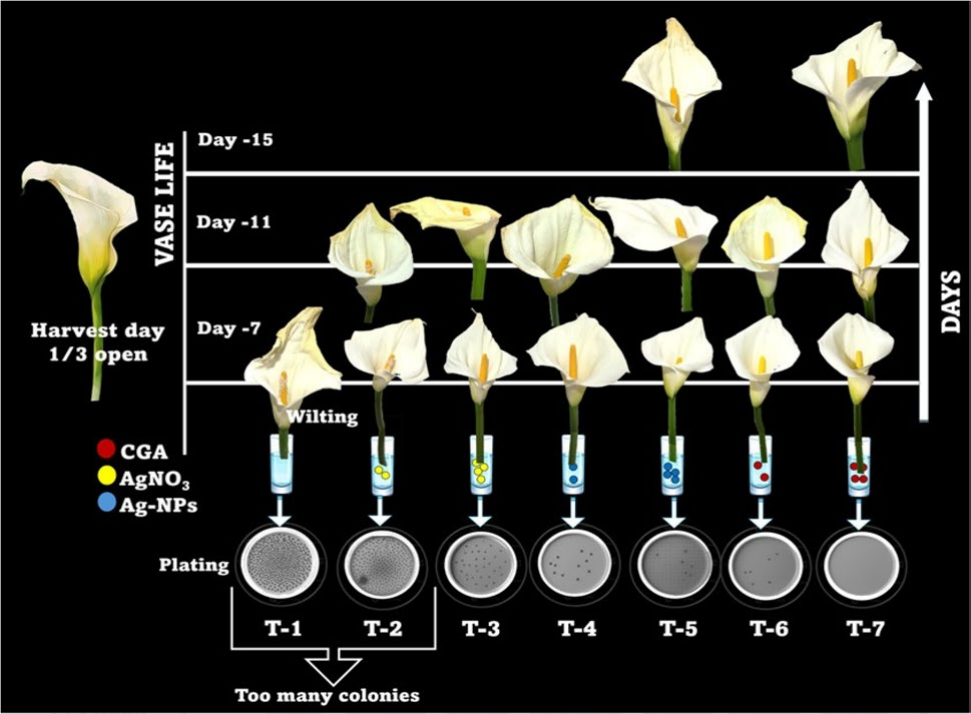
Effect of optimal levels of silver nitrate, silver nanoparticles, and chlorogenic acid on the appearance of cut *Z. aethiopica* flowers over 7, 11, and 15 days. Treatments: T1 = Control; T2 = AgNO₃ 50 mg L⁻¹; T3 = AgNO₃ 100 mg L⁻¹; T4 = AgNPs 15 mg L⁻¹; T5 = AgNPs 30 mg L⁻¹; T6 = CGA 15 mg L⁻¹; T7 = CGA 30 mg L⁻¹. The images represent three independent replicates (*n* = 3)

Data presented in Figs. 4 and 5 showed that silver nitrate, silver nanoparticles, and chlorogenic acid significantly affected the vase life of *Z. aethiopica* cut flowers within each season (*p ≤* 0.05). The higher concentration across all treatments resulted in significantly longer vase life than the lower concentration.

The untreated control exhibited the shortest vase life (7.33 and 6.00 days in the first and second seasons, respectively). Silver nitrate (AgNO₃) significantly extended vase life relative to the control (*p ≤* 0.05), reaching 11.33–12.00 days in the first season and 11.00–11.67 days in the second season. Silver nanoparticles (AgNPs) produced a greater promotional effect than AgNO₃, particularly at the higher concentration (T5), prolonging vase life to 15.00 and 14.67 days in the first and second seasons, respectively. Chlorogenic acid (CGA) treatments also significantly improved vase life (*p ≤* 0.05), with the highest concentration (T7) achieving the maximum values recorded in this study (16.33 and 16.00 days in the first and second seasons, respectively). Hence, the higher concentration of CGA exhibited the most pronounced and statistically significant effect, followed by high-concentration AgNPs, demonstrating their superior efficacy in delaying senescence and enhancing postharvest quality of calla cut flowers.

### Water relationship

#### Relative Fresh Weight (RFW%)

**Fig. 6:**
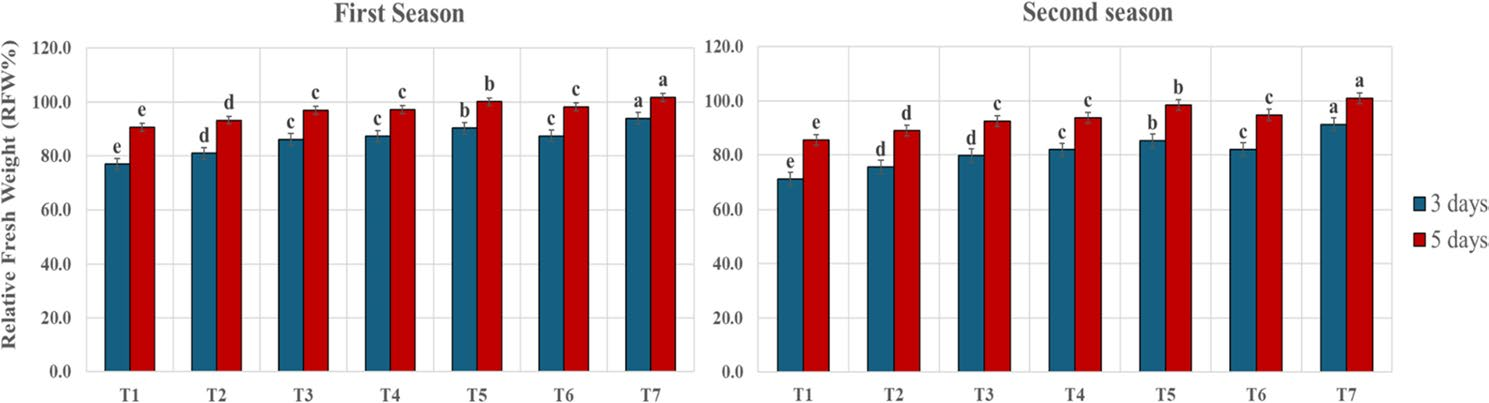
Relative fresh weight (RFW%) of Z. aethiopica cut flowers treated with silver nitrate (AgNO₃), silver nanoparticles (AgNPs), and chlorogenic acid (CGA) after 3 and 5 days during two experimental seasons. Bars represent means ± SE (n = 3) for each season, analyzed separately. Different letters indicate significant differences among treatments within each season according to Duncan’s multiple range test at p ≤ 0.05. Treatments: T1 = Control; T2 = AgNO₃ 50 mg L⁻¹; T3 = AgNO₃ 100 mg L⁻¹; T4 = AgNPs 15 mg L⁻¹; T5 = AgNPs 30 mg L⁻¹; T6 = CGA 15 mg L⁻¹; T7 = CGA 30 mg L⁻¹

The effect of different concentrations of silver nitrate (AgNO₃), silver nanoparticles (AgNPs), and chlorogenic acid (CGA) on the relative fresh weight (RFW%) of Z. aethiopica cut flowers during the first and second experimental seasons is presented in Fig.6. After 3 days, the highest RFW% was recorded in T7 (CGA 30 mg L⁻¹) with 93.92% and 91.35% in the first and second seasons, respectively, followed by T5 (AgNPs 30 mg L⁻¹) with 90.25% and 85.36%. Over 5 days, RFW% continued to increase, with T7 maintaining the highest values (101.70% and 101.02%), followed by T5 (100.10% and 98.48%). AgNO₃ treatments (T2: 50 mg L⁻¹ and T3: 100 mg L⁻¹) significantly improved RFW% compared to the control (T1), achieving 86.07–79.72% at 3 days and 96.94–92.50% at 5 days (p ≤ 0.05, Duncan’s test). T4 (AgNPs 15 mg L⁻¹) and T6 (CGA 15 mg L⁻¹) also significantly increased RFW%, but the values were lower than those for T5 and T7. The control cut flowers (T1) consistently exhibited the lowest RFW%, with 76.85% and 71.15% at 3 days, and 90.60% and 85.60% at 5 days, in the first and second seasons, respectively.

#### Floral water uptake (g flower⁻¹ day⁻¹)

**Fig. 7:**
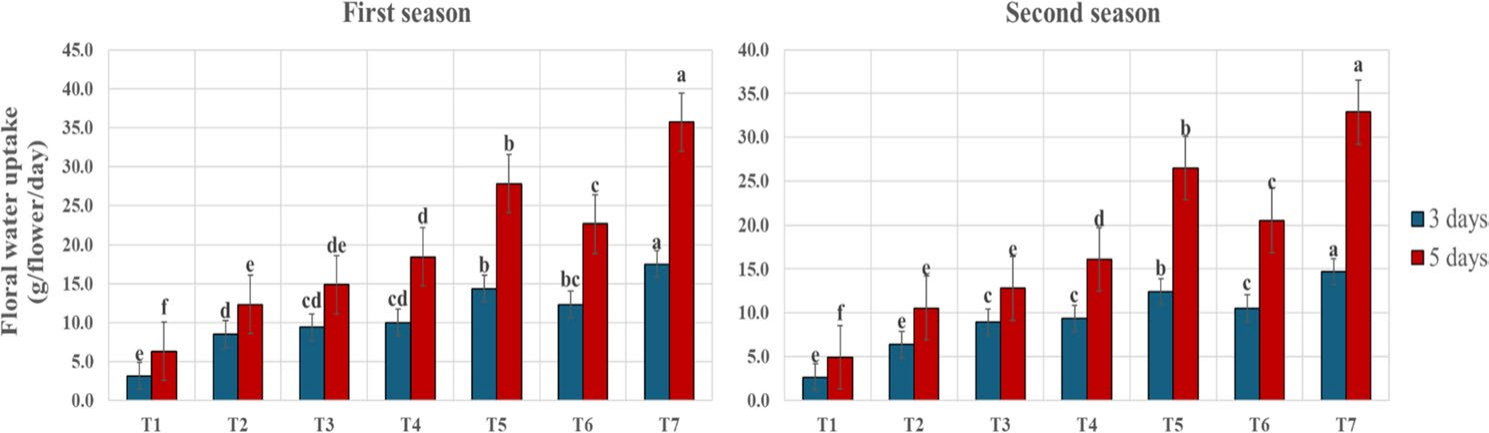
Water uptake of *Z. aethiopica* cut flowers treated with silver nitrate (AgNO₃), silver nanoparticles (AgNPs), and chlorogenic acid (CGA) after 3 and 5 days during two experimental seasons. Bars represent means ± SE (*n* = 3) for each season, analyzed separately. Different letters indicate significant differences among treatments within each season according to Duncan’s multiple range test at *p* ≤ 0.05. Treatments: T1 = Control; T2 = AgNO₃ 50 mg L⁻¹; T3 = AgNO₃ 100 mg L⁻¹; T4 = AgNPs 15 mg L⁻¹; T5 = AgNPs 30 mg L⁻¹; T6 = CGA 15 mg L⁻¹; T7 = CGA 30 mg L⁻¹

The floral water uptake rate of Z. aethiopica cut flowers increased progressively after three and five days in response to the applied concentrations of silver nitrate, silver nanoparticles, and chlorogenic acid (Fig. 7). All treatments significantly enhanced water uptake compared with the untreated control (p ≤ 0.05, Duncan’s test). After 3 days, the control (T1) showed the lowest uptake values (3.14 and 2.65 g flower⁻¹ day⁻¹ in the first and second seasons, respectively). Treatments containing AgNO₃ (T2 and T3) resulted in moderate increases, ranging from 8.46 to 9.38 g flower⁻¹ day⁻¹ during the first season and 6.36 to 8.94 g flower⁻¹ day⁻¹ during the second season. Higher uptake values were recorded in AgNP treatments (T4 and T5), ranging from 10.00 to 14.34 g flower⁻¹ day⁻¹ in the first season and from 9.33 to 12.40 g flower⁻¹ day⁻¹ in the second season. CGA treatments (T6 and T7) further increased water uptake, with T7 exhibiting the highest values (17.49 and 14.73 g flower⁻¹ day⁻¹ for the first and second seasons, respectively). After 5 days, the same trend continued. The control flowers (T1) again recorded the lowest uptake (6.27 and 4.93 g flower⁻¹ day⁻¹). AgNO₃ treatments produced values between 12.29 and 14.83 g flower⁻¹ day⁻¹, whereas AgNP treatments increased uptake to 18.43–27.78 g flower⁻¹ day⁻¹ in the first season and 16.11–26.46 g flower⁻¹ day⁻¹ in the second season. CGA treatments showed marked improvements, with T7 maintaining the highest uptake (35.68 and 32.87 g flower⁻¹ day⁻¹).

#### Floral water loss (g flower⁻¹ day⁻¹)

**Fig. 8:**
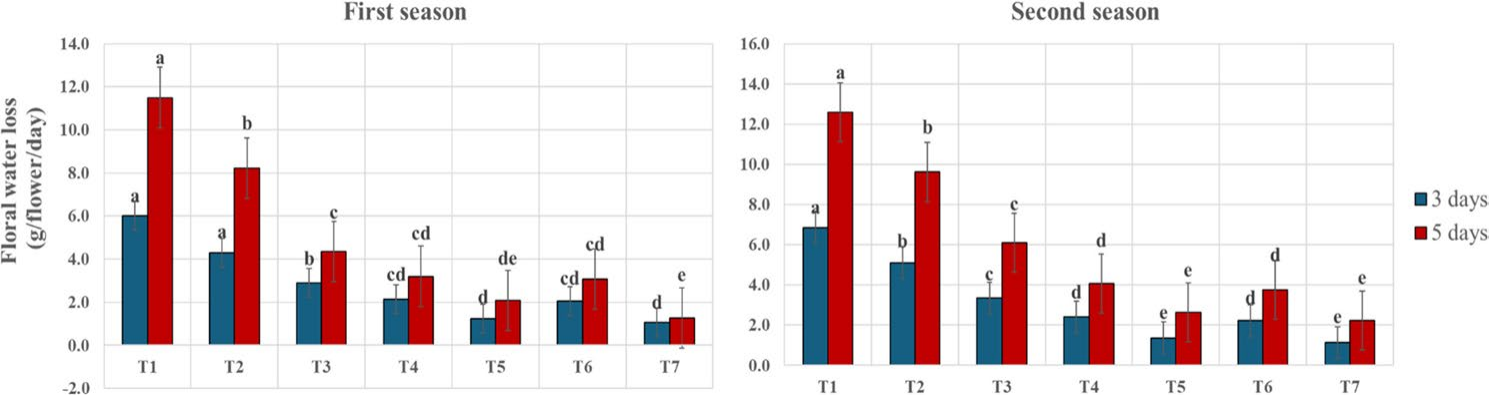
Water loss of *Z. aethiopica* cut flowers treated with silver nitrate (AgNO₃), silver nanoparticles (AgNPs), and chlorogenic acid (CGA) after 3 and 5 days during two experimental seasons. Bars represent means ± SE (*n* = 3) for each season, analyzed separately. Different letters indicate significant differences among treatments within each season according to Duncan’s multiple range test at *p* ≤ 0.05. Treatments: T1 = Control; T2 = AgNO₃ 50 mg L⁻¹; T3 = AgNO₃ 100 mg L⁻¹; T4 = AgNPs 15 mg L⁻¹; T5 = AgNPs 30 mg L⁻¹; T6 = CGA 15 mg L⁻¹; T7 = CGA 30 mg L⁻¹

Data presented in Fig. 8 show the effect of silver nitrate (AgNO₃), silver nanoparticles (AgNPs), and chlorogenic acid (CGA) on the floral water loss of Z. aethiopica cut flowers after 3 and 5 days during the first and second experimental seasons. All treatments significantly reduced water loss compared with the untreated control (p ≤ 0.05, Duncan’s test). After 3 days, the highest water loss values were recorded in the control treatment (T1), reaching 6.00 and 6.86 g flower⁻¹ day⁻¹ in the first and second seasons, respectively. AgNO₃ treatments (T2 and T3) significantly decreased water loss to values ranging between 4.28 and 2.90 g flower⁻¹ day⁻¹ in the first season and 5.11–3.34 g flower⁻¹ day⁻¹ in the second season. AgNP treatments (T4 and T5) further reduced water loss, with values ranging from 2.14 to 1.24 g flower⁻¹ day⁻¹ (first season) and 2.41 to 1.37 g flower⁻¹ day⁻¹ (second season). CGA treatments (T6 and T7) also resulted in significant reductions, with T7 recording the lowest water-loss values after 3 days (1.06 and 1.13 g flower⁻¹ day⁻¹ in the first and second seasons, respectively). After 5 days, a similar trend was observed. The control treatment (T1) exhibited the highest water loss (11.50 and 12.58 g flower⁻¹ day⁻¹), while AgNO₃ treatments showed reduced values (8.22–4.36 g flower⁻¹ day⁻¹ and 9.61–6.09 g flower⁻¹ day⁻¹ in the first and second seasons, respectively). AgNPs treatments produced further reductions (3.20–2.08 g flower⁻¹ day⁻¹ and 4.07–2.64 g flower⁻¹ day⁻¹). CGA treatments continued to lower water loss, with T7 showing the minimum values (1.27 and 2.22 g flower⁻¹ day⁻¹).

### Chemical analysis

#### Enhancement of physiological and biochemical attributes of *Z. aethiopica* Cut flowers by silver nitrate, silver nanoparticles, and chlorogenic acid

**Fig. 9:**
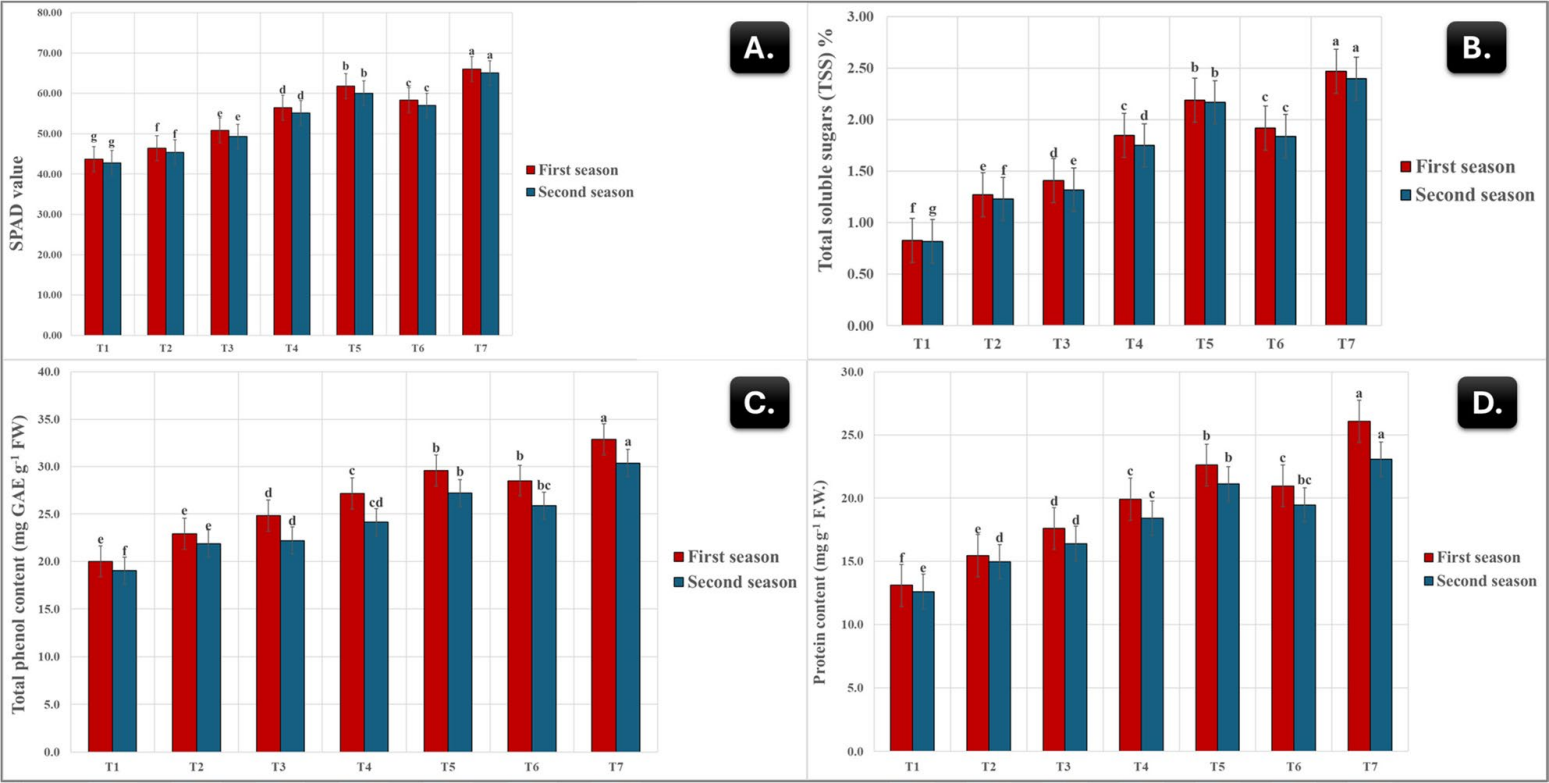
Relative chlorophyll index (SPAD), total soluble sugars (TSS, %), total phenol content (mg GAE g⁻¹ FW), and protein content (mg g⁻¹ FW) of Z. aethiopica cut flowers treated with silver nitrate (AgNO₃), silver nanoparticles (AgNPs), and chlorogenic acid (CGA) during two experimental seasons. **A** SPAD value; (**B**) Total soluble sugars (TSS, %); (**C**) Total phenol content (%); (**D**) Total soluble protein content (mg g⁻¹ FW). Bars represent means ± SE (n = 3) for each season, analyzed separately. Different letters indicate significant differences among treatments within each season according to Duncan’s multiple range test at p ≤ 0.05. Treatments: T1 = Control; T2 = AgNO₃ 50 mg L⁻¹; T3 = AgNO₃ 100 mg L⁻¹; T4 = AgNPs 15 mg L⁻¹; T5 = AgNPs 30 mg L⁻¹; T6 = CGA 15 mg L⁻¹; T7 = CGA 30 mg L⁻¹

The application of silver nitrate (AgNO₃), silver nanoparticles (AgNPs), and chlorogenic acid (CGA) significantly improved both physiological and biochemical attributes of Z. aethiopica cut flowers over two consecutive growing seasons (p ≤ 0.05) as shown in Fig.9A, B, C and D. Relative chlorophyll index (SPAD; Fig. 9A) and total soluble sugars (TSS, %; Fig. 9B) were markedly enhanced with increasing concentrations of all treatments. CGA 30 mg L⁻¹ produced the highest SPAD value (66.05 in the first season and 65.08 in the second season; +51.0% and + 51.9% relative to the control, respectively) and TSS (2.47% and 2.40%; +197.6% and + 192.7%), followed by AgNPs 30 mg L⁻¹ (61.81 and 60.06 SPAD; +41.3% and + 40.3%; 2.19% and 2.17% TSS; +163.9% and + 164.6%, respectively). Control consistently showed the lowest values (43.72 and 42.80 SPAD; 0.83% and 0.82% TSS). Although numerical values in the second season were generally slightly lower than in the first, the treatment response pattern remained consistent across both seasons, while the trends among treatments remained consistent.

Similarly, the total phenol content (mg GAE g⁻¹ FW) (Fig. 9C) and protein content (mg g⁻¹ FW; Fig. 9D) were significantly increased by the treatments. Flowers treated with CGA 30 mg L⁻¹ exhibited the highest phenol (32.88 and 30.38 mg GAE g⁻¹ FW; +64.3% and +59.7% in the first and second seasons, respectively) and total soluble protein content (26.09 and 23.08 mg g⁻¹ FW; +99.4% and +83.3% in the first and second seasons, respectively), followed by AgNPs 30 mg L⁻¹ (29.60 and 27.21 mg GAE g⁻¹ FWphenol; +47.7% and +43.0%; 22.62 and 21.12 mg g⁻¹ FW soluble protein; +72.7% and +67.7%, in the first and second seasons, respectively). The lowest phenol (20.02 and 19.02 mg GAE g⁻¹ FW) and soluble protein (13.09 and 12.59 mg g⁻¹ FW) were obtained from the control. These results collectively highlight the potent role of CGA and AgNPs in enhancing both the physiological and biochemical quality of cut calla lily flowers, underscoring their potential to extend vase life and improve postharvest performance, as shown in Fig.15.

#### Enhancement of antioxidant enzyme activities of *Z. aethiopica* cut flowers by silver nitrate, silver nanoparticles, and chlorogenic acid

**Fig. 10:**
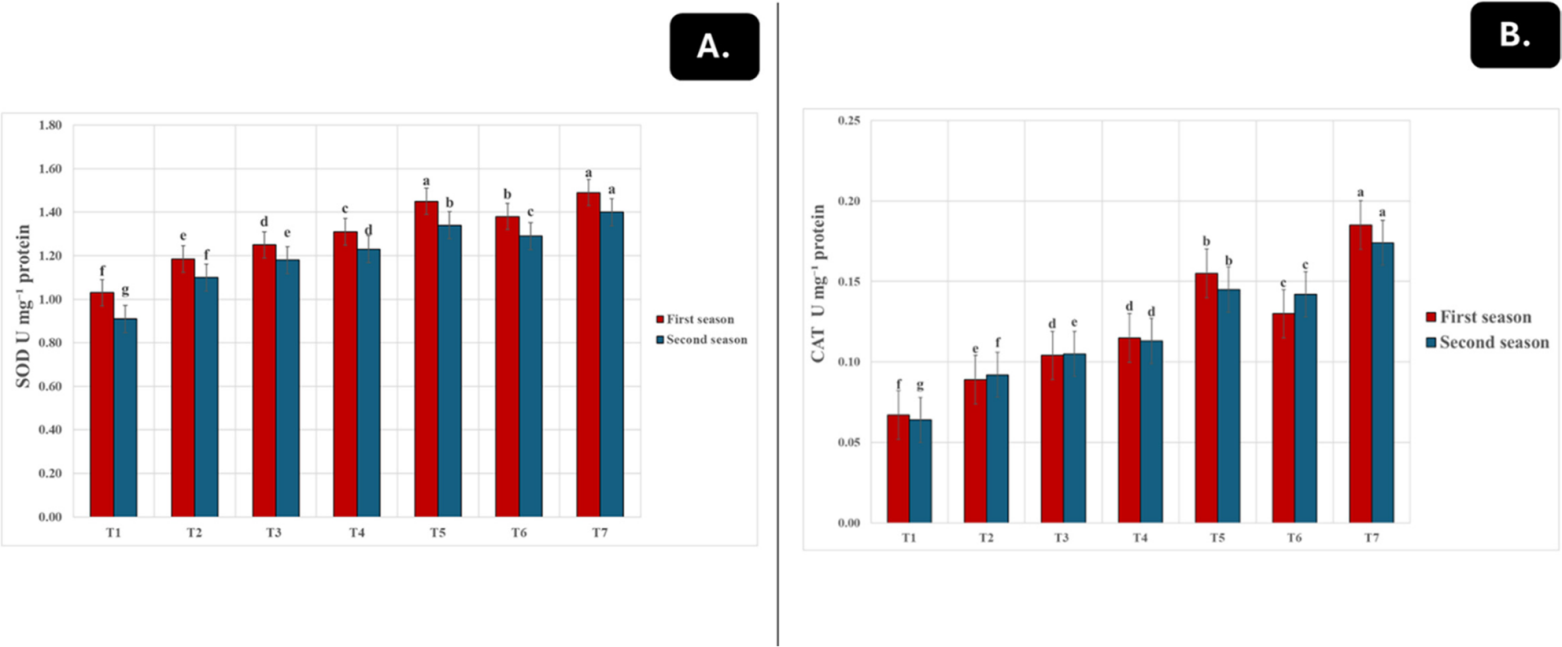
Catalase (CAT, U mg⁻¹ protein) and superoxide dismutase (SOD, U mg⁻¹ protein) activities of Z. aethiopica cut flowers treated with silver nitrate (AgNO₃), silver nanoparticles (AgNPs), and chlorogenic acid (CGA) during two experimental seasons. **A** Superoxide dismutase (SOD, U mg⁻¹ protein); (**B**) Catalase (CAT, U mg⁻¹ protein). Bars represent means ± SE (n = 3) for each season, analyzed separately. Different letters indicate significant differences among treatments within each season according to Duncan’s multiple range test at p ≤ 0.05. Treatments: T1 = Control; T2 = AgNO₃ 50 mg L⁻¹; T3 = AgNO₃ 100 mg L⁻¹; T4 = AgNPs 15 mg L⁻¹; T5 = AgNPs 30 mg L⁻¹; T6 = CGA 15 mg L⁻¹; T7 = CGA 30 mg L⁻¹

The effect of treatments was significant for SOD (A) and CAT (B) activities during both seasons (p ≤ 0.05, Fig 10). Compared with the control (T1), all treatments significantly increased CAT and SOD activities. The highest activities were observed under the highest concentration of chlorogenic acid (T7: CGA 30 mg L⁻¹), with CAT reaching 0.185 and 0.174 U mg⁻¹ protein (+176.1% and +171.9%) and SOD reaching 1.49 and 1.40 U mg⁻¹ protein (+44.7% and +53.8%) in the first and second seasons, respectively.

Chlorogenic acid at the lower concentration (T6: 15 mg L⁻¹) also enhanced enzyme activities (0.130 and 0.142 U mg⁻¹ protein CAT, +94.0% and +121.9%; 1.38 and 1.29 U mg⁻¹ protein SOD, +34.0% and +41.8%), showing intermediate effects between AgNPs and the control. High AgNPs(T5: 30 mg L⁻¹) increased CAT to 0.155 and 0.145 U mg⁻¹ protein (+131.3% and +126.6%) and SOD to 1.45 and 1.34 U mg⁻¹ protein (+40.8% and +47.3%), while lower AgNPs (T4: 15 mg L⁻¹) showed moderate enhancements (0.115 and 0.113 U mg⁻¹ protein CAT, +71.6% and +76.6%; 1.31 and 1.23 U mg⁻¹ protein SOD, +27.2% and +35.2%). AgNO₃ treatments also improved enzyme activities in a dose-dependent manner: T2 (50 mg L⁻¹) increased CAT to 0.089 and 0.092 U mg⁻¹ protein (+32.8% and +43.8%) and SOD to 1.185 and 1.10 U mg⁻¹ protein (+15.0% and +20.9%), whereas T3 (100 mg L⁻¹) reached 0.104 and 0.105 U mg⁻¹ protein CAT (+55.2% and +64.1%) and 1.25 and 1.18 U mg⁻¹ protein SOD (+21.4% and +29.7%).

#### Reducing oxidative stress in *Z. aethiopica* cut flowers using silver nitrate, silver nanoparticles, and chlorogenic acid

**Fig. 11:**
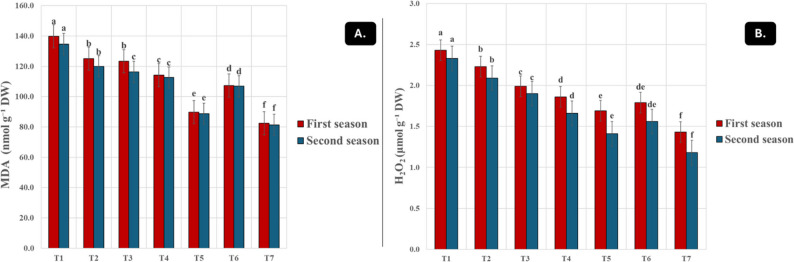
Malondialdehyde (MDA) and hydrogen peroxide (H₂O₂) contents of Z. aethiopica cut flowers treated with silver nitrate (AgNO₃), silver nanoparticles (AgNPs), and chlorogenic acid (CGA) during two experimental seasons. **A** MDA content (nmol g⁻¹ DW); (**B**) H₂O₂ content (μmol g⁻¹ DW). Bars represent means ± SE (n = 3) for each season, analyzed separately. Different letters indicate significant differences among treatments within each season according to Duncan’s multiple range test at p ≤ 0.05. Treatments: T1 = Control; T2 = AgNO₃ 50 mg L⁻¹; T3 = AgNO₃ 100 mg L⁻¹; T4 = AgNPs 15 mg L⁻¹; T5 = AgNPs 30 mg L⁻¹; T6 = CGA 15 mg L⁻¹; T7 = CGA 30 mg L⁻¹

Significant differences were observed among treatments for MDA (A; nmol g⁻¹ DW) and H₂O₂ (B; μmol g⁻¹ DW) in Z. aethiopica cut flowers during both seasons (p ≤ 0.05, Figure 11). Control flowers (T1) showed the highest oxidative stress, with MDA of 0.140 and 0.135 nmol g⁻¹ DW and H₂O₂ of 2.43 and 2.33 μmol g⁻¹ DW in the first and second seasons, respectively. All treatments significantly reduced MDA and H₂O₂ compared to the control. The greatest effect was observed with the highest concentration of chlorogenic acid (T7: CGA 30 mg L⁻¹) as in Fig.11 and 15, with MDA of 0.083 and 0.081 nmol g⁻¹ DW (−40.7% and −40.0%) and H₂O₂ of 1.43 and 1.18 μmol g⁻¹ DW (−41.2% and −49.4%). High AgNPs (T5: 30 mg L⁻¹) also markedly reduced oxidative markers, with MDA of 0.090 and 0.089 nmol g⁻¹ DW (−35.7% and −34.1%) and H₂O₂ of 1.69 and 1.41 μmol g⁻¹ DW (−30.5% and −39.5%). Moderate reductions were observed with lower AgNPs (T4: 15 mg L⁻¹) and lower chlorogenic acid (T6: CGA 15 mg L -¹), while AgNO₃ treatments (T2: 50 mg L⁻¹, T3: 100 mg L⁻¹) showed smaller, dose-dependent decreases. Specifically, T2 had MDA of 0.125 and 0.120 nmol g⁻¹ DW (−10.7% and −11.1%) and H₂O₂ of 2.23 and 2.09 μmol g⁻¹ DW (−8.2% and −10.3%), whereas T3 recorded MDA of 0.123 and 0.116 nmol g⁻¹ DW (−12.1% and −14.1%) and H₂O₂ of 1.99 and 1.90 μmol g⁻¹ DW (−18.1% and −18.5%).

### Mean bacterial counts (CFU mL⁻¹)

#### Reduction of bacterial growth in vase solution of *Z. aethiopica* cut flowers by silver nitrate, silver nanoparticles, and chlorogenic acid

**Fig. 12:**
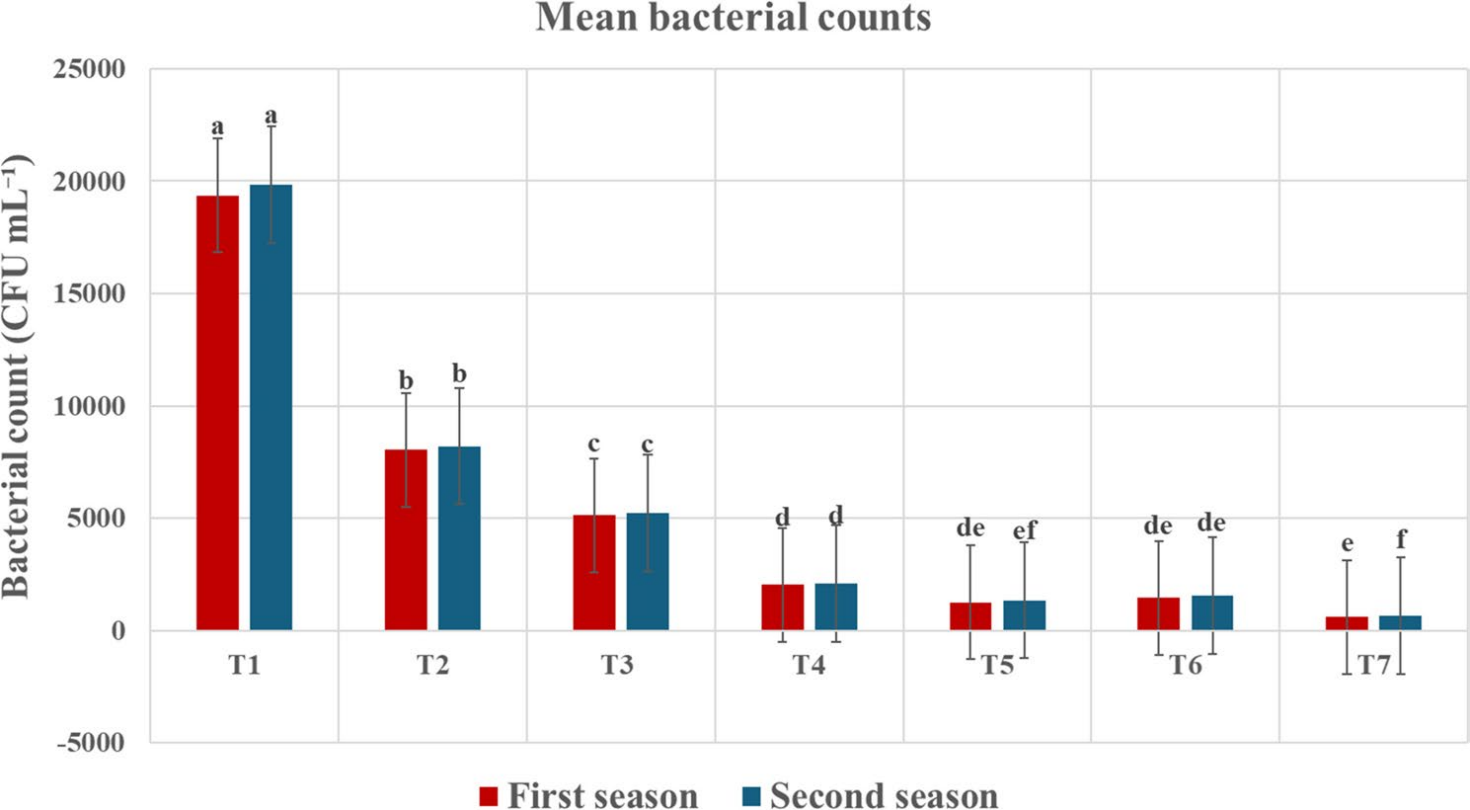
Mean bacterial counts (CFU 100 mL⁻¹) in vase solutions of *Z. aethiopica* cut flowers treated with silver nitrate (AgNO₃), silver nanoparticles (AgNPs), and chlorogenic acid (CGA) during two experimental seasons. Bars represent means ± SE (*n* = 3) for each season, analyzed separately. Different letters indicate significant differences among treatments within each season according to Duncan’s multiple range test at *p ≤* 0.05. Treatments: T1 = Control; T2 = AgNO₃ 50 mg L⁻¹; T3 = AgNO₃ 100 mg L⁻¹; T4 = AgNPs 15 mg L⁻¹; T5 = AgNPs 30 mg L⁻¹; T6 = CGA 15 mg L⁻¹; T7 = CGA 30 mg L⁻¹

Microbial growth in vase solutions is a major factor affecting the postharvest life and quality of cut flowers. Therefore, treatments that suppress bacterial growth are crucial to extend the vase life of cut flowers. In this study, the effect of silver nitrate (AgNO₃), silver nanoparticles (AgNPs), and chlorogenic acid (CGA) on bacterial populations in the vase solution of *Z. aethiopica* cut flowers was evaluated during two consecutive growing seasons (Fig. 12). Significant differences were observed among treatments for mean bacterial counts (CFU 100 mL^− 1^) (*p ≤* 0.05). The highest bacterial populations were recorded in control (T1: distilled water), with 19,350 and 19,850 CFU 100 mL^− 1^ in the first and second seasons, respectively. All treatments significantly reduced bacterial growth compared with the control.

The highest concentration of chlorogenic acid (T7: CGA 30 mg L⁻¹) had the greatest antimicrobial effect, resulting in lower counts of 600 and 650 CFU 100 mL⁻¹, representing reductions of − 96.9% and − 96.7% compared to the control, respectively. High AgNPs (T5: 30 mg L⁻¹) also effectively inhibited bacterial growth (1,250 and 1,350 CFU 100 mL⁻¹, − 93.5% and − 93.2%), followed by lower chlorogenic acid (T6: CGA 15 mg L⁻¹, 1,450 and 1,550 CFU 100 mL⁻¹, − 92.5% and − 92.2%) and lower AgNPs (T4: 15 mg L⁻¹, 2,025 and 2,100 CFU 100 mL⁻¹, − 89.5% and − 89.4%). Silver nitrate treatments (T2: 50 mg L⁻¹ and T3: 100 mg L⁻¹) showed moderate reductions, with T2 at 8,050 and 8,200 CFU 100 mL⁻¹ (− 58.4% and − 58.6%) and T3 at 5,125 and 5,225 CFU 100 mL⁻¹ (− 73.5% and − 73.7%).

### Scanning Electron Microscopy (SEM)

**Fig. 13:**
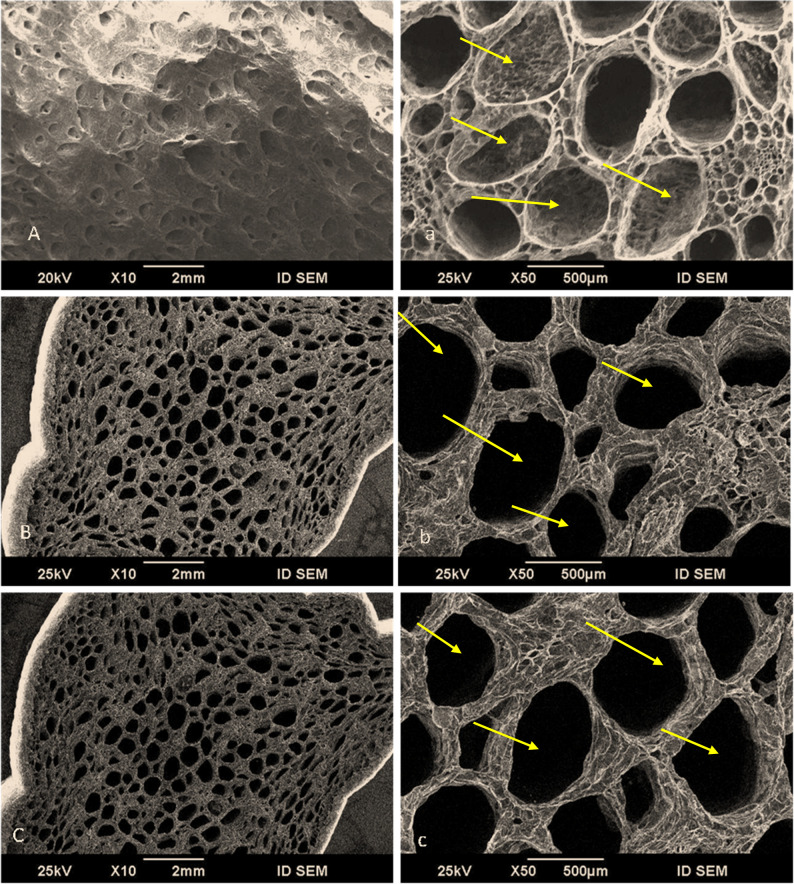
Scanning electron microscopy (SEM) micrographs of xylem vessels in the stem bases of *Zantedeschia aethiopica* cut flowers showing bacterial proliferation and vessel blockage under different treatments. **A** Control (distilled water); (**B**) AgNPs 30 mg L⁻¹; (**C**) Chlorogenic acid 30 mg L⁻¹. The images represent three independent replicates. Scale bars: 2 mm (**A**–**C**) and 500 μm (a–c)

The xylem morphology at the stem bases of *Z. aethiopica* cut flowers was examined using SEM to assess the effects of silver nanoparticles (T5: AgNPs 30 mg L⁻¹) and chlorogenic acid (T7: CGA 30 mg L⁻¹) compared with untreated flowers (T1: control) (Fig. 13). In control flowers, the xylem vessels exhibited severe blockages with bacterial accumulation along the vessel walls (Fig. 13A, a), which can impede water transport and contribute to early wilting of flowers and leaves.

Flowers treated with T5 (AgNPs 30 mg L⁻¹) showed substantially clearer xylem vessels with reduced bacterial presence, although minor bacterial clusters and partial obstruction were still observed (Fig. 13B, b). Similarly, flowers treated with T7 (CGA 30 mg L⁻¹) displayed wide-open vessels with minimal bacterial accumulation and less xylem blockage compared with the control (Fig. 13C, c). Generally, both treatments improved xylem integrity and facilitated water movement, but complete elimination of microbial presence was not achieved.

### Correlation, PCA, and path analysis of postharvest traits

**Fig. 14:**
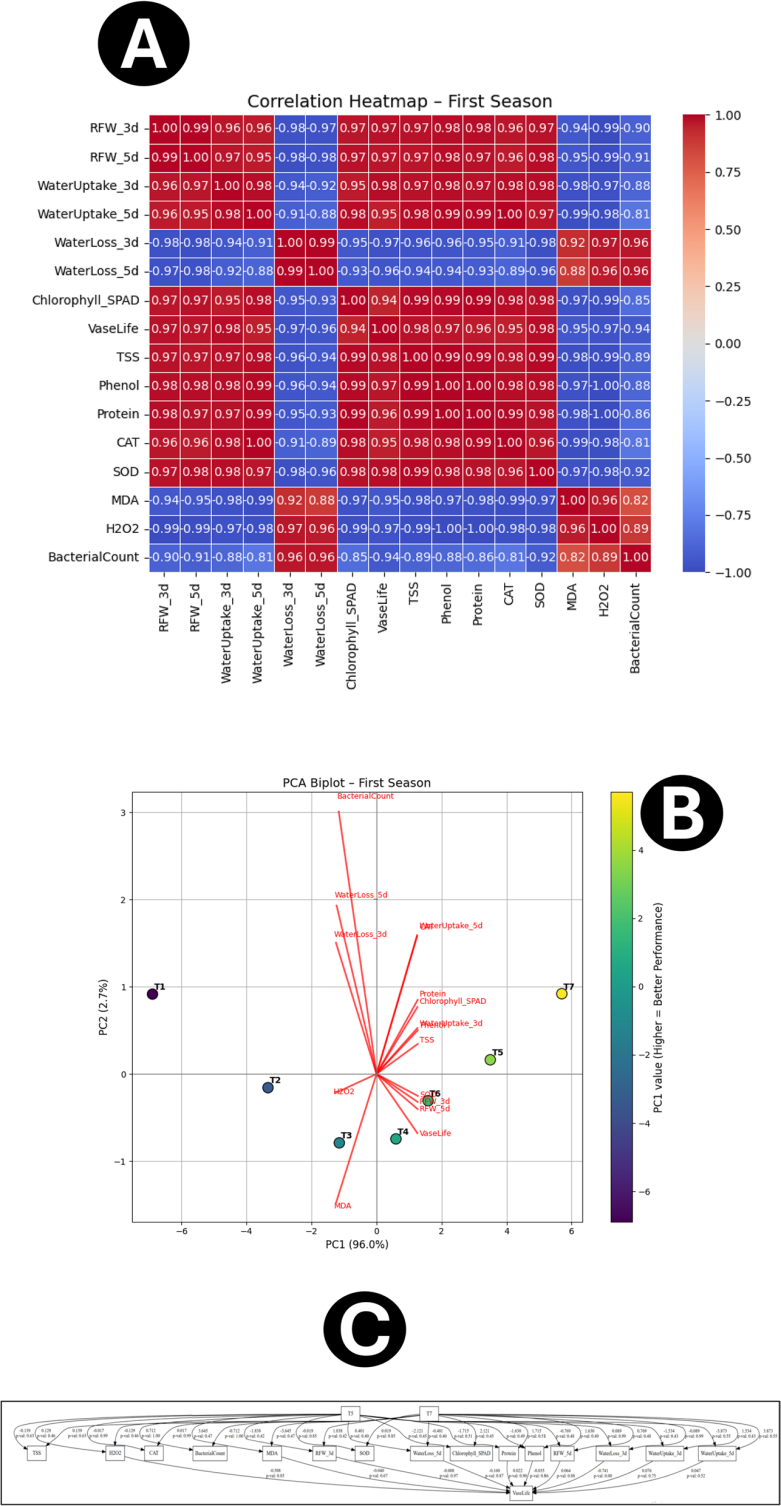
Heatmap of correlations among physiological and morphological traits (**A**), principal component analysis (PCA) biplot illustrating the contribution of traits and treatments to overall variability (**B**), and path analysis diagram showing the direct and indirect effects of measured traits on vase life of cut *Zantedeschia aethiopica* flowers (**C**). The data represent three independent replicates (*n* = 3)

The correlation heatmap (Fig. 14A) demonstrated strong positive correlations among postharvest quality parameters of cut *Z. aethiopica* flowers. Vase life was highly correlated (*r* > 0.90) with relative fresh weight (RFW), water uptake, and relative chlorophyll index (SPAD), while phenolic and protein contents also exhibited positive associations. In contrast, oxidative stress markers (H₂O₂ and MDA) were negatively associated with vase life. Positive correlations were depicted in red, while negative correlations were depicted in blue, with color intensity indicating the strength of the association.

Treatments with AgNPs (T5) and chlorogenic acid (T7) enhanced water uptake and mitigated oxidative damage, resulting in extended vase life compared to the control (T1) and AgNO₃ treatments (T2 and T3).

The PCA biplot (Fig. 14B) indicated that the first two principal components explained most of the variance. PC1 was positively associated with vase life, RFW, water uptake, and SPAD value, whereas H₂O₂ and MDA were negatively associated. Treatments involving AgNPs and chlorogenic acid clustered on the positive side of PC1, underscoring their effectiveness in maintaining water balance and delaying senescence. Path analysis (Fig. 14C) revealed that water uptake and RFW had the strongest positive direct effects on vase life, followed by phenolic content and relative chlorophyll index (SPAD) retention, whereas oxidative stress markers had negative effects. AgNPs and chlorogenic acid improved vase life indirectly by improving water relations and lowering oxidative stress, highlighting the importance of combining antioxidant and hydration strategies to extend postharvest longevity.

### Graphical summary of postharvest responses in cut *Zantedeschia aethiopica*

**Fig. 15:**
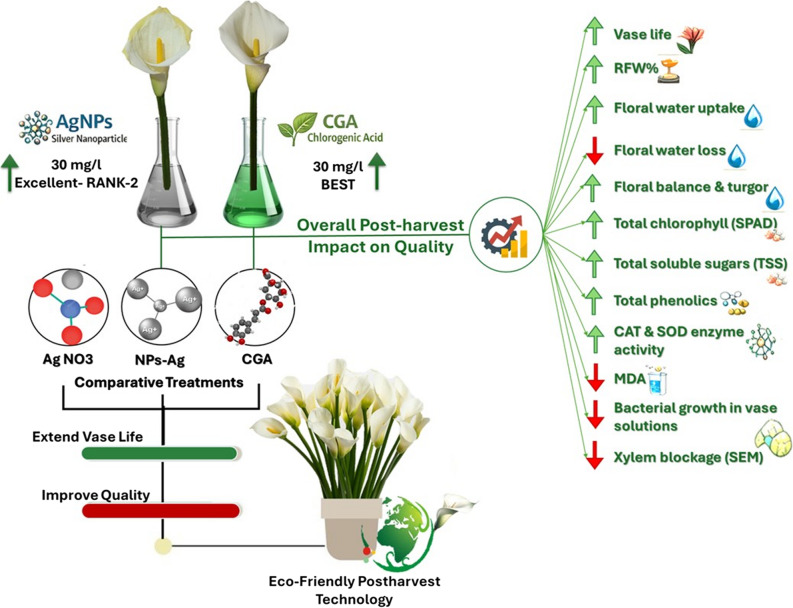
Graphical summary of the overall postharvest responses of cut *Zantedeschia aethiopica* flowers to AgNO₃, silver nanoparticles (AgNPs), and chlorogenic acid (CGA) treatments. CGA at 30 mg L⁻¹ exhibited the greatest improvement in flower quality, followed by AgNPs at the same concentration. The data represent three independent replicates (*n* = 3)

As shown in Fig. 15, both AgNPs and chlorogenic acid (CGA) markedly improved vase life and key quality attributes, as evidenced by increased relative fresh weight (RFW%), enhanced water uptake, improved water balance and turgor, and higher chlorophyll content (SPAD). In addition, these treatments significantly increased total soluble sugars (TSS) and total phenolic content, and stimulated antioxidant enzyme activities (CAT and SOD). In contrast, significant reductions were observed in water loss, malondialdehyde (MDA) accumulation, bacterial proliferation in vase solutions, and xylem vessel blockage. These responses indicate improved membrane stability, reduced oxidative damage, and enhanced hydraulic conductivity. Collectively, the results demonstrate that CGA and AgNPs act as effective, eco-friendly postharvest treatments that prolong vase life and preserve flower quality by coordinating the regulation of water relations, antioxidant defense systems, and microbial inhibition.

## Discussion

Utilizing bio-stimulants in vase solutions strengthens the antibacterial and antioxidant defense mechanisms of cut flowers and modulates postharvest physiological processes. These procedures enhance floral quality and vase life. Silver ions (Ag⁺), released from AgNO₃ and silver nanoparticles (AgNPs), exhibit potent antibacterial activity. They disrupt bacterial membrane proteins by interacting with sulfhydryl (–SH) groups, leading to microbial cell death [44]. Additionally, Ag⁺ inhibits ethylene action by delaying floral senescence and binding to its receptors [45]. Furthermore, silver nitrate is a common preservative, but its toxicity and cost limit its practical use [46, 47].

Nanotechnology is an important recent development with numerous applications in agriculture.

Bio-stimulants are crucial for increasing crop productivity and also play a significant role in postharvest applications by improving flower quality and reducing microbial activity [48, 49]. In contrast, AgNPs offer a sustainable alternative with enhanced biocidal efficacy at lower concentrations, reduced toxicity, and minimized environmental impact. Their nanoscale size facilitates penetration of bacterial cell walls, and their large surface area intensifies electrostatic interactions that inhibit microbial growth in vase solutions and stem ends, preserving xylem function and water uptake [50]. Additionally, the controlled release of Ag⁺ ions helps regulate water balance and delay senescence, due to the combined effect of suppressing both water stress and ethylene damage [51]. In our study, silver nitrate (AgNO_₃_), silver nanoparticles (AgNPs), and chlorogenic acid (CGA) dramatically improved the vase life of calla cut flowers. By boosting relative fresh weight (RFW%) and promoting increased water uptake, these treatments also significantly reduced water loss compared to control cut flowers, as shown in Figs. 4, 4 and 7, and 8. The results of this study align with findings [52, 53] that ethylene sensitivity in cut flowers is closely associated with ACS activity and variations in ethylene receptor levels. AgNPs also suppress ethylene responses in cut flowers by inhibiting ACS activity and preventing ethylene binding to its receptors. This effect is attributed to the release of Ag⁺ ions, which inhibit ethylene perception by replacing the Cu²⁺ cofactor at receptor sites. They also interfere with ethylene biosynthesis by downregulating ACS and ACO gene expression, delaying flower senescence [54]. Meanwhile, chlorogenic acid (CGA), a natural polyphenol, enhances postharvest flower quality through its antioxidant and regulatory functions. It scavenges reactive oxygen species (ROS), inhibits lipid peroxidation, and maintains cellular redox balance, thus reducing oxidative stress and delaying senescence [18]. CGA also lowers ethylene production by suppressing cellular metabolic and catabolic activities. Ilea et al. [17] reported that CGA (50 mg L⁻¹) inhibited the enzyme involved in the conversion of malate to pyruvate and NADPH, which are key substrates in respiratory metabolism, contributing to reduced metabolic activity and improved cellular energy balance in treated tissues. Furthermore, the reduced ethylene levels observed in CGA-treated cut calla lily flowers may result from decreased microbial activity, since pathogens can produce ethylene during growth [55]. Although both CGA and silver-based treatments show promise individually, their combined effects on cut flower preservation remain unexplored [56].

Investigating this potential synergy could provide innovative strategies to extend vase life and enhance the marketability of ornamental flowers [57, 58].

Water relations, including relative flower weight and water uptake, serve as essential indicators for monitoring senescence and evaluating the effectiveness of vase solutions in prolonging the longevity of cut flowers. Detachment from the mother plant disrupts water uptake while transpiration continues, leading to water stress and accelerated senescence [59]. Optimal ornamental value is maintained when water uptake exceeds the transpiration loss [9, 60]. Both Ag⁺ treatments and CGA, particularly at higher concentrations, improved water balance, spathe size, and relative fresh weight. CGA outperformed AgNPs and conventional preservatives such as AgNO₃, underscoring its potential as a sustainable and eco-friendly alternative. Control flowers displayed rapid senescence symptoms, such as spadix abscission, wilting, and spathe browning, whereas AgNPs and CGA treatments preserved turgidity and semi-open spathe morphology for longer periods. AgNO_₃_ exhibited only moderate effects, initially delaying senescence but later accelerating degradation, indicating that its efficacy depends on concentration and formulation. These findings align with [35, 44], who reported that AgNPs extended vase life more effectively than bulk AgNO_₃_, due to improved antimicrobial action, ethylene inhibition, and maintenance of xylem conductivity [51, 61]. Similarly, CGA delays senescence by inhibiting ethylene biosynthesis, respiration, and ROS accumulation, while enhancing antioxidant defenses and stabilizing membranes, ultimately promoting improved water retention [16, 17]. It activates phenylpropanoid enzymes (CAT, SOD, POD, PAL, C4H, CAD, PPO), promoting the synthesis of lignin, phenolics, and suberin, which reinforce vascular tissues and facilitate wound healing [61–63]. Despite its multifunctionality, CGA remains underutilized in postharvest floriculture.

In the present study, CGA and AgNPs significantly improved the water relations of cut calla lilies (Fig. 7). These findings align with [56], who reported that CGA reduces water loss and delays dehydration by promoting membrane stabilization, thereby preserving tissue integrity during storage.

Additionally, recent studies suggest that CGA helps to maintain water balance in plant tissues by stabilizing membranes, reducing permeability, and limiting water loss. As a potent antioxidant, it protects membrane integrity, lowers respiration rates, and enhances chlorophyll content and vitamin C levels, improving water retention and reducing water consumption in asparagus during storage [64]. On the other hand, Bayanati et al. [65] reported that AgNPs significantly enhanced relative fresh weight, membrane stability, and vase life in cut rose flowers without exhibiting toxicity.

The current study demonstrates that the application of CGA, AgNPs, and AgNO_3_ significantly increases photosynthetic capacity, phenolic content, and enzyme activity, while decreasing H₂O₂ and MDA (Figs. 9 and 10, and 11). These results agree with [52], who demonstrated that the application of AgNPs in the vase solution effectively prolonged vase life in carnations by improving water balance and enhancing antioxidant activity. Hence, beyond its antioxidant role, CGA also acts as an effective physiological regulator and a promising natural alternative to chemical preservatives.

During the senescence of cut flowers, membrane degradation and vacuolar collapse increase cellular permeability, leading to electrolyte leakage and subsequent loss of turgor pressure. These changes further contribute to cell wall disintegration and disrupt metabolic processes. Consequently, vital cellular components, such as amino acids, sugars, phenolic compounds, and vitamins, are depleted. This compromises cellular homeostasis, increases susceptibility to pathogens, and accelerates the onset of visible symptoms such as wilting, chlorosis, and tissue breakdown [66, 67]. At the same time, malondialdehyde (MDA), a product of lipid peroxidation, serves as a reliable indicator of membrane damage. These compounds are used as markers to assess the severity of stress and the effectiveness of treatments, such as nanoparticles or antioxidants, indicating their role in mitigating oxidative damage in plants. Our results showed that CGA and AgNPs significantly reduced malondialdehyde (MDA) and hydrogen peroxide (H₂O₂) levels, markers of lipid peroxidation and oxidative stress, with CGA being more effective. Elevated H₂O₂ triggers oxidative damage by attacking membrane lipids, increasing MDA, and disrupting membrane integrity [68]. CGA’s potent ROS-scavenging capacity helps stabilize membranes and mitigate oxidative stress, consistent with previous reports in postharvest asparagus and peach fruit [16, 64]. The CGA application significantly increased phenolic content, thereby maintaining redox homeostasis and mitigating oxidative damage under stress, likely through upregulation of genes involved in antioxidant defense and phenolic biosynthesis pathways [61]. Moreover, the accumulation of osmoprotectants, such as phenolics, soluble sugars, and proteins, in CGA-treated tissues plays a vital role in cellular protection, osmotic adjustment, and postharvest stress resilience [17, 56]. However [19, 69], reported that CGA is a polyphenolic molecule that can donate electrons to neutralize free radicals, lowering H₂O₂ generation and preventing lipid peroxidation cascades that lead to MDA formation.

Moreover, CGA may inhibit ROS-producing pathways (such as NADPH oxidases or mitochondrial electron leakage), thereby reducing oxidative stress in the first place [70]. Additionally, Nguyen et al. [70] reported that a decrease in ROS (H₂O₂) and damage markers (MDA) suggests that CGA helps maintain redox balance, preventing a shift toward a pro-oxidant state rather than reacting to damage. Furthermore, reduced oxidative damage provides functional evidence of protection in our study, highlighting the significance of elevated enzyme activities, such as SOD, CAT, and APX. Reduced MDA and H₂O₂ indicate that these enzymatic defenses, possibly strengthened by CGA, are successfully preventing oxidative damage [71]. Under floral stress conditions, the activities of key antioxidant enzymes such as SOD, which catalyzes the dismutation of superoxide radicals into H₂O₂, and CAT, which decomposes H₂O₂ into water and oxygen, are actively regulated to mitigate oxidative damage [72]. This enzymatic regulation is a vital component of the intrinsic defense system in cut flowers, where ROS accumulate during postharvest storage and accelerate senescence and quality deterioration. CGA has been shown to enhance these enzymatic antioxidant defenses.

As shown in Fig. 15 and the heatmap analysis in Fig. 14, these findings suggest that CGA and AgNPs can enhance vase life, water retention, physiological and biochemical traits, and protective enzyme levels in calla cut flowers, thereby promoting their overall health and longevity in the current study. These results were confirmed by Mei et al. [66], who reported that CGA application significantly increased SOD and CAT activities, nearly doubling their levels compared to untreated controls. This result is consistent with our findings in CGA-treated cut calla lily flowers. This biochemical enhancement was associated with a marked reduction in H₂O₂ accumulation, indicating that CGA directly mitigates oxidative stress. Consistent with these observations, our results showed a significant upregulation of SOD and CAT activities in CGA-treated flowers. In addition, the reduced levels of H₂O₂ and malondialdehyde (MDA) in treated flowers provide additional evidence that CGA mitigates oxidative damage by maintaining cellular redox balance.

Controlling microbial growth after harvest is crucial for maintaining floral quality and extending the vase life of cut flowers. Microbial contamination often blocks xylem vessels through microbial aggregation and/or secretions, impairing water uptake, accelerating wilting, and ultimately reducing the commercial value of the flowers [13, 14]. In this context, our findings demonstrate that chlorogenic acid (CGA) effectively suppresses microbial proliferation and enhances the physiological sustainability of cut Zantedeschia (calla lily) flowers (Figs. 12 and 13). This effect is primarily attributed to CGA’s strong antioxidant capacity and its ability to disrupt microbial cell walls and interfere with key cellular functions, inhibiting bacterial and fungal growth [16]. Notably, this study is the first scientific report on CGA as a postharvest treatment for Zantedeschia, highlighting its potential as a natural, safe alternative in the cut flower industry. AgNPs, on the other hand, exhibit broad-spectrum antimicrobial activity via multiple mechanisms, including membrane penetration, DNA disruption, and ROS induction, which trigger oxidative stress in microbial cells [50]. Due to their ultrafine particle size (< 100 nm), AgNPs exhibit unique physicochemical properties that distinguish them from conventional silver forms, thereby enhancing their antimicrobial efficacy [51, 72, 73]. However, concerns remain regarding the standalone use of AgNPs, as their long-term persistence in the environment may lead to heavy metal accumulation and potential ecological and health risks [48]. Among the tested treatments, CGA and AgNPs consistently outperformed AgNO₃ in maintaining water balance, providing antimicrobial activity, and reducing oxidative stress, delaying senescence. The superior efficacy of CGA emphasizes its promise as a natural, eco-friendly preservative with minimal environmental impact. Collectively, these findings suggest that combining plant-derived bioactive compounds, such as CGA, with nanotechnology-based agents, such as AgNPs, provides a synergistic and sustainable approach to postharvest management. Such strategies could significantly improve longevity, aesthetic appeal, and marketability of cut flowers while aligning with global efforts toward environmentally responsible floriculture.

## Conclusion

The present study highlights the differential effects of silver nitrate (AgNO_₃_), silver nanoparticles (AgNPs), and chlorogenic acid (CGA) on the postharvest performance of cut calla lily flowers. Silver nanoparticles (AgNPs) were highly effective in prolonging vase life, maintaining water balance, and enhancing antioxidant activity, while reducing oxidative stress markers, such as hydrogen peroxide (H₂O₂) and malondialdehyde (MDA). Chlorogenic acid (CGA) efficiently suppressed microbial growth in both the vase solution and stem ends, increased relative fresh weight (RFW%), and enhanced the antioxidant defense, thereby delaying senescence. Silver nitrate (AgNO₃) showed moderate preservative effects, initially delaying senescence but with limited long-term efficacy compared to AgNPs and CGA.

The optimal preservative concentrations were 30 mg L⁻¹ for CGA and 30 mg L⁻¹ for AgNPs, whereas AgNO_₃_ was effective at 100 mg L⁻¹ but to a lesser extent. Overall, CGA and AgNPs represent promising eco-friendly alternatives to conventional chemical preservatives, offering distinct physiological and antimicrobial benefits when applied individually. These findings provide valuable guidance for the targeted use of natural and nanotechnology-based preservatives in the cut flower industry. 

## Data Availability

The datasets used and/or analyzed during the present investigation available from the corresponding author on reasonable request.
